# Head and Neck Metastatic Tumors: a Retrospective Survey of Iranian Patients

**Published:** 2015-03

**Authors:** Donia Sadri, Arash Azizi, Sareh Farhadi, Hojjat Shokrgozar, Navid Entezari

**Affiliations:** a Dept. of Oral and Maxillofacial Pathology, Islamic Azad University, Dental Branch of Tehran, Tehran, Iran.; b Dept. of Oral Medicine, Islamic Azad University, Dental Branch of Tehran, Tehran, Iran.; c Private Dentist, Tehran, Iran.

**Keywords:** Oral Cancer, Head and Neck, Metastasis

## Abstract

**Statement of the Problem:**

The head and neck region is an uncommon site for metastatic involvement, but it can be the first and only symptom of primary cancer. The incidence of these tumors and their primary origins are limited in Iranian patients.

**Purpose:**

Therefore, this retrospective study aimed to investigate the frequency and the common related clinical manifestations, as well as, the most common types of cancers and the prevalent sites of the primary tumor.

**Materials and Method:**

All medical records related to patients with history of head and neck tumors between 1991 and 2011 at Iran Cancer Institute were evaluated and the essential information was statistically analyzed.

**Results:**

Sixty cases of cervical lymph node metastasis (0.36%) and 26 cases of head and neck metastatic tumors (0.16%) including 17 cases of distant cancer (0.10%) were recorded among all 16232 registered cancers. Out of all distant head and neck metastatic tumors, 4 cases were related to oral and maxillofacial area. Pain, swelling of neck, oral mucosa ulcer and dryness were the chief complaints. Squamous cell carcinoma and adenocarcinoma were the most frequent types of cancers. The most common metastatic sites were cervical musculature, scalp and parotid gland, and the most prevalent sites of primary tumor in females were breast and lung in males.

**Conclusion:**

According to these cases, the incidence rate of head and neck metastatic tumors seems to be low. However, feasible similarity of clinical presentation of oral metastatic lesions to benign lesions might result in misdiagnosis. Hence, biopsy is mandatory in any case with unusual clinical presentation, especially in patients with a known malignant disease.

## Introduction

Metastatic tumors have notable diagnostic and also therapeutic importance in head and neck, especially in the oral and maxillofacial region, because they can be the first evidence for the spread of primary malignant cells from other organs. [1] Yet, there is not any precise epidemiological information about them. [2] A retrospective study (2000) reported 80000 people dying out of oral primary and metastatic cancer, 58000 cases of whom resided in less-developed countries. [3]

Metastasis is identified as spread of cancer from one part of the body to another. The metastatic tumor contains cells similar to original ones. In this regard, distant metastasis refers to cancer that is spread from the original (primary) tumor to distant organs or distant lymph nodes. [4]

Metastatic tumors are often detected after recognizing the primary ones; but sometimes oral metastatic lesion is the first symptom of a malignant tumor. [1] Oral metastatic tumors are found in the spread of malignant cells that affect another organ and are transferred through lymphatic or vascular systems. [5] Unfortunately, oral metastatic disease is usually manifestation of an advanced disease; hence, an unknown number of patients die without investigation for occult oral metastases. [6]

The actual incidence of these cancers is unknown. Approximately 1-3% of all malignant oral neoplasms [7-8] and 1% of all types of cancers [9-11] have been reported to have affected both soft and hard tissues. Lung cancer has been considered as the most common metastasis occurring in the oral soft tissue in males, followed by kidney, liver and prostate cancers, as well as malignant melanoma. On the other side, the most prevalent metastasis in females is breast cancer, followed by genital, lung and kidney malignancies. [12-13]

Metastasis in majority of sarcomas occurs through the vascular system and carcinomas through the lymphatic system. Seemingly, the presence of metastatic tumors in oral soft tissue is related to vascular ones. Although the jaws are uncommon sites for hard tissue metastasis, 80% of them have been detected on the lower jaw. [14-16] Recent studies have reported the prevalence of gnathic bone metastasis to be 20 times more than the oral soft tissue. Gingiva and the tongue are respectively the most common soft tissue sites involved with metastasis. [13] The most common cancer metastasis to lip and tongue are introduced as thyroid and esophagus cancers. [14-16]

Limited information is available about the head and neck metastatic tumors, especially oral and maxillofacial ones and their primary origins in Iranian patients. Moreover, changes have been observed in the incidence and mortality rate of cancer types over time in this population. So, this retrospective study was carried out in Iran Cancer Institute to evaluate these tumors in terms of frequency, primary origins, common related clinical manifestations, and the most common types of cancers, as well as the most common sites of the primary tumor. 

## Materials and Method

The current study evaluated all medical records of patients with history of head and neck tumors that were registered at the Pathology Department of Iran Cancer Institute, Tehran University of Medical Sciences between 1991 and 2011. The samples which had definite pathologic diagnosis and complete medical and demographic information were chosen and studied. The essential information related to samples including gender, age, site of metastases and primary tumor, date of examination, any habit and the patients’ chief complain were extracted from information submitted on biopsy requisition. The retrieved data were registered on the study information form. Finally, the frequencies of variables were reported using SPSS software, version 16. 

## Results

A total number of 16232 cancer cases were registered at Iran Cancer Institute from 1991 to 2011, with 60 cases of cervical lymph node metastasis (0.36%) and 26 cases of head and neck metastatic tumors (0.16%), including 17 cases of distant cancer (0.10%), 4 out of which were related to oral and maxillofacial area. Out of those 17 cases of head and neck metastatic tumors, 9 were male (52.9%) and 8 were female (47.1%), with the mean±SD age of 49.7±1. The main complaints of the patients were reported as pain and swelling of the neck accompanied by mucosal ulcer and dryness. 

Table 1 represents the frequency of the studied head and neck metastatic tumors based on the type of cancers, indicating squamous cell carcinoma and adenocarcinoma as the most common cancers.

**Table 1:** Frequency of head and neck metastatic tumors at Iran Cancer Institute (1991-2011) according to the type of cancer

**Type of cancer**	**Frequency**	**%**
Squamous cell carcinoma	7	41
Adenocarcinoma	5	29.5
Melanoma	2	11.8
Lobular Carcinoma	2	11.8
Papillary Carcinoma	1	5.9
Total	17	100

Frequency of the studied head and neck metastatic tumors according to the site of metastasis has been demonstrated in Table 2. The most common involved site was cervical musculature, followed by scalp and parotid gland. The frequency of the studied oral and maxillofacial metastatic tumors with respect to site of primary tumor has been shown in Table 3; according to which, the most commonly involved sites in females were breast, and lung in males.

**Table 2:** Frequency of head and neck metastatic tumors at Iran Cancer Institute (1991-2011) based on the site of metastasis

**Site of metastasis**	**Frequency**	**%**
Cervical musculature	5	29.2
Scalp	4	23.6
Parotid gland	2	11.8
Mandible bone	1	5.9
Buccal mucosa	1	5.9
Tongue	1	5.9
Larynx	1	5.9
Sub-mandibular salivary gland	1	5.9
Brain	1	5.9
Total	17	100

**Table 3:** The frequency of head and neck metastatic tumors at Iran Cancer Institute (1991-2011) with respect to the site of the primary tumor

**Site of the primary tumor**	**Frequency**	**%**
Unknown	4	23.6
Breast	3	17.4
Skin (except head and neck area)	2	11.8
Lung	2	11.8
Lung Thyroid gland	1	5.9
Buccal mucosa	1	5.9
Esophageous	1	5.9
Larynx	1	5.9
Ovary	1	5.9
Pancreases	1	5.9
Total	17	100

Information related to the four cases of oral and maxillofacial metastatic tumors is represented in Table 4. Based on this table, the most common cancer type was SCC, the prevalent primary sites were breast and larynx, and the metastasized sites were sub-mandibular salivary gland, tongue, buccal mucosa, and mandible bone.

**Table 4:** Information about the oral and maxillofacial metastatic tumors at Iran Cancer Institute (1991-2011)

**No.**	**Primary origin**	**Type of cancer**	**Age**	**Sex**	**Site of metastatic tumor**
1	Unknown	SCC	56	Male	Sub-mandibular salivary gland
2	Larynx	SCC	65	Male	Buccal mucosa
3	Breast	Lobular carcinoma	26	Female	Mandible bone
4	Unknown	SCC	26	Male	Tongue

## Discussion

The results of this study revealed that distant head and neck metastatic tumors constituted 0.1% of all registered cancers over 20 years. Kumar *et al.* reported 1-3% of all malignant oral neoplasms, [7] and the studies by Butter *et al.*, D’Silva *et al.* and Lim *et al.* reported 1% of all type of cancers as oral and maxillofacial metastatic tumors. [9-10, 14] More studies with greater sample size such as the one performed by D’Silva *et al.* [8] reflecting a 45-year evaluation can explain the difference between the incidences of these metastatic tumors.

Focusing on primary tumor, the most common sites were breast in females and lung in males, which is in line with the Chinese study that reported the lung and breast as the most common sites of primary tumors. [15] Whereas, Lim *et al.* demonstrated liver, lung and thyroid gland, [10] and the studies by D’Silva *et al.* and Butter *et al.* revealed the lung, breast and prostate gland as the most common sites for primary tumor. [9, 14] The related epidemiological parameters such as racial and demographic information might be different. [16] Also, the types of malignancies that frequently metastasize to this region reflect the relative incidence of cancers in a definite population at a definite time. [12]

In the present study, the most common metastatic sites were cervical musculature, followed by scalp and parotid gland respectively. The mandible was reported as the most common metastatic site in the studies by Daley *et al.* and Butter *et al.* These studies had focused only on the oral cavity and jaw bones and the cervical area was excluded. So, the difference in the obtained results might be related to the different sites studied. [12, 14] However, the results found by Bonder *et al.* regarding the frequency of metastatic site were similar to that of the current study. [8]

Lim *et al.* reported the mean age of patients to be 55.5 years old, while this number was 60 in the study conducted by Butter *et al.* and Daley *et al.* [10, 12, 14] Like the present study, Butter *et al.* demonstrated equal proportion of male to female patients; [14] however, the study by Lim *et al.* calculated the ratio to be 1.9:1. Explaining the different results, type of metastatic cancers in males and females is not the same in different geographic regions. [10]

Similar to the present study, Hirshberg *et al.* reported that the patients mainly complained of swelling and cellulites, [13] but Bonder *et al.* reported other signs such as lower lip and chin paresthesia. [8] The high frequency of metastatic tumor on the anterior part of the mandible seems to be related to these differences. Furthermore, Bonder *et al.* detected that oral metastatic signs were the first complaints of unknown primary malignancy for 1 of the eight studied cases. [8] Butter *et al.* found one third of oral metastatic tumors as the first signs of primary cancer. Both of the above mentioned studies have degrees of similarities with the present study. [14]

The actual incidence of metastatic disease in the head and neck region is unclear because of the presence of occult lesions, undiagnosed mass, or the radiolucencies in these sites and misdiagnosis of lesions which may present insufficient medical knowledge. The histological appearance of metastatic head and neck tumor is often poorly differentiated, and it makes it challenging to determine the location of the primary lesion. Therefore, taking a thorough medical history can facilitate the diagnosis; however, conducting a screening by using a panel of immunohistochemical stains may lead to diagnosis. [9] In addition to the population incidence, data from the current study revealed the relative frequency of types of cancers that metastasize to these regions, reflecting the incidence of these cancers in Iranian population.

## Conclusion

Compared to other worldwide studies, metastatic head and neck tumors were rare at Iran Cancer Institute between 1991 and 2011. Most common carcinoma type cancers were squamous cell carcinoma and adenocarcinoma with pain and swelling of neck as their most frequent signs. Cervical musculature was the most commonmetastasized sites. Also, the most common site of primary tumor was breast for females and lung for males. Evaluating these cases revealed that the clinical presentation of a metastatic lesion in the head and neck- especially oral and maxillofacial region- may lead to a misdiagnosis of a benign process. Thus, biopsy is mandatory in any case with unusual clinical presentation, especially those with a known malignant disease. 
